# Supplementary material for: The information theory of developmental pruning: Optimizing global network architectures using local synaptic rules
Source: PLoS Comput Biol. 2021 Oct 11;17(10):e1009458. doi: 10.1371/journal.pcbi.1009458 (PMC8584672; doi:10.1371/journal.pcbi.1009458)
Supplement: S1 Text — (PDF) [file pcbi.1009458.s001.pdf]

# The information theory of developmental pruning: Optimizing global network architectures using local synaptic rules

## S1: Latent activity may indicate optimal model size

We observed that the classification error increased simultaneously with the average hidden unit activity in $\mathbf{h}^2$ (see Figure A). However, this effect was specific to models pruned according to criteria that removed whole neurons from the model (i.e. our FI estimates and random unit removal to a lesser extent). For the other pruning criteria the average activity in $\mathbf{h}^2$ remained sparse and did not increase during the course of pruning. The effect is most pronounced for models pruned according to our variance estimate of FI. Here, the performance drop matches a sudden increase in mean hidden layer activity after the third pruning iteration.

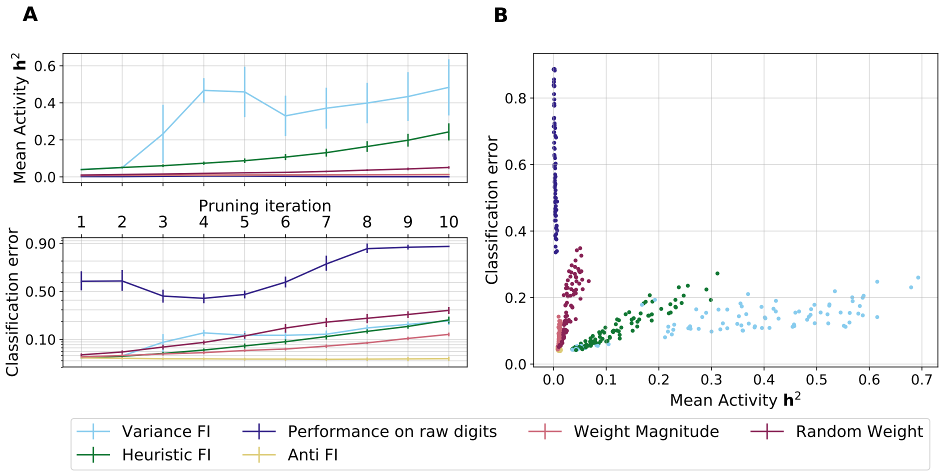


**Fig A. Latent activity increased with classification error.**
The average hidden unit activity tended to increase with the classification error for models that lost whole units as a consequence of synapse pruning. (A) Average activity in $\mathbf{h}^2$ and classification error over the course of 10 pruning iterations. All data points are averages from 10 independent simulations, and error bars denote one standard deviation. (B) Classification error vs. average activity in $\mathbf{h}^2$.

We hypothesize that the hidden activity may be a suitable indicator of the optimal model size and encode a stop signal for pruning. At the same time this suggests that the detrimental effects of repeated pruning could potentially be attenuated by a homeostatic adjustment of the parameters which would account for the loss of synapses by down-regulating the activity of the remaining neurons.
