# Supplementary material for: The information theory of developmental pruning: Optimizing global network architectures using local synaptic rules
Source: PLoS Comput Biol. 2021 Oct 11;17(10):e1009458. doi: 10.1371/journal.pcbi.1009458 (PMC8584672; doi:10.1371/journal.pcbi.1009458)
Supplement: S2 Text — (PDF) [file pcbi.1009458.s002.pdf]

The information theory of developmental pruning: Optimizing global network architectures using local synaptic rules

## S2: Comparison after 10 pruning iterations

One of our main results is that FI pruning typically removes all incoming or outgoing connections to a hidden unit, such that synapse pruning can effectively implement neuron pruning. A secondary effect of this is a faster model reduction: when a unit lost all its inputs *or* targets, all its remaining weights were also removed. That is why pruning criteria that failed to remove neurons (i.e. weight magnitude and random weight pruning) needed more iterations of pruning to reach a comparable number of remaining weights. In Figures 3 and 4 of the main manuscript we presented the results for a comparable number of remaining weights across criteria. For completeness, we present the results after the same number of 10 pruning iterations for each criterion in Figures A and Figures B.

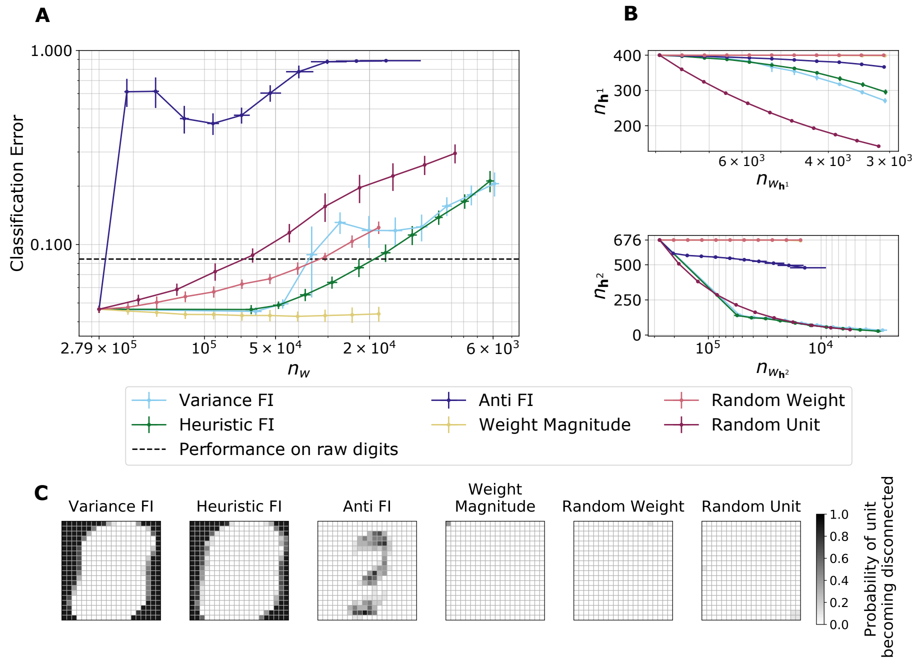


**Fig A. Encoding performance during the course of 10 pruning iterations for each pruning criterion.**
(A) Classification error of a logistic regression classifier trained on $\mathbf{h}^2$ encodings as a function of remaining weights $n_w$. The dotted line stands for the baseline error of a classifier trained on the raw digits. All data points are averages from 10 independent simulations, and error bars denote one standard deviation. (B) Number of latent units in $\mathbf{h}^1$ and $\mathbf{h}^2$ as a function of remaining weights over the course of ten iterations of pruning. (C) Final visible layer connectivity after pruning for 10 iterations according to different criteria. The probability of a unit being disconnected is shown in gray-scale, with black denoting units that were disconnected in all simulations.

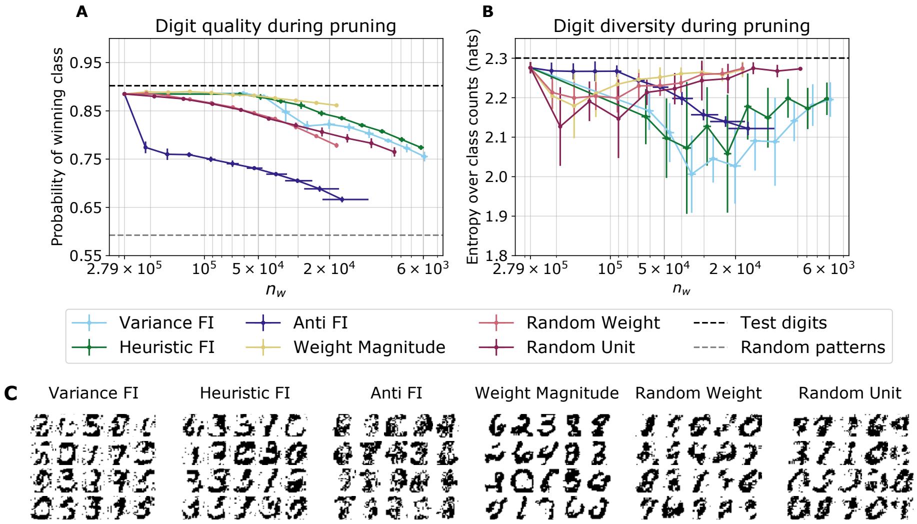


**Fig B. Generative performance during the course of 10 pruning iterations for each pruning criterion.**

(A) Maximum class probability assigned to generated samples from pruned networks after 10 iterations of pruning, averaged over 10 runs. This summarizes the confidence of a classifier that the generated digits belonged to a specific class. Error bars denote standard deviations. The black and gray dashed line show the confidence of the classifier for the MNIST test digits and randomly generated patterns, respectively. (B) Entropy over the distribution of generated digits. An entropy value of ≈ 2.30 nats corresponds to even coverage of all digits, which is achieved by the test digits (dotted line). All data points are averages from 10 independent simulations, and error bars denote one standard deviation. (C) Examples of generated patterns after pruning completed. Note the different numbers of remaining weights per criterion when comparing the samples.
