# Supplementary material for: The information theory of developmental pruning: Optimizing global network architectures using local synaptic rules
Source: PLoS Comput Biol. 2021 Oct 11;17(10):e1009458. doi: 10.1371/journal.pcbi.1009458 (PMC8584672; doi:10.1371/journal.pcbi.1009458)
Supplement: Attachment — Submitted filename: rebuttal_letter1_ploscomp.pdf [file pcbi.1009458.s003.pdf]

Reply to the Reviewers

Re: Manuscript ID PCOMPBIOL-D-20-02149
"The Information Theory of Developmental Pruning: Optimizing Global Network Architecture Using Local Synaptic Rules"
Carolin Scholl, Michael E. Rule, and Matthias H. Hennig
PLOS Computational Biology

Dear Reviewers and Editors,

Thank you for the consideration of our manuscript and the possibility to resubmit a draft for publication in PLOS Computational Biology. We appreciate the time and effort that you dedicated to providing feedback and are grateful for the detailed comments on and valuable improvements to our paper.

We have substantially revised the manuscript in light of the reviewer feedback and editorial guidance. We highlighted all changes, with blue indicating added content and red indicating deleted content. Please see a point-by-point response to the reviewers' comments and concerns below. All page and line numbers refer to the revised manuscript file.

We hope that the revised manuscript will remedy all concerns, but are happy to consider further revisions, and we thank you for your continued interest in our research.

Yours sincerely,
the authors

Reviewer #1, comment #1
The paper investigates the pruning of connections with low Fisher Information in restricted and deep Boltzmann machines. This approach is compared with pruning based on a locally computable proxy of Fisher information, the maximum Fisher information, and the synaptic weight, as well as random pruning. As the most interesting property, the authors demonstrate that the low FI approach typically removes all connections to a hidden neuron, such that the synapse pruning can also implement neuron pruning. At the same time, the pruned networks retain high encoding/classification and generative performance. The methods the authors use to demonstrate their finding are sound and appropriate. However, I am missing error estimates or a validation by multiple trials.

Moreover, although the topic of pruning is a very interesting and relevant topic both in biological and artificial neural networks, I think the findings and writing of the paper are more on the artificial intelligence than on the biological networks side (usage of RBM, training on MNIST/CIFAR, no comparison to experimental data), which should be changed to be suitable for publication in this journal.

Our response #1.1
Regarding the proximity to machine learning, we partially agree. Theoretical neuroscience often uses "models" with unclear connections to the biology for mathematical convenience. We made several changes to the paper to better highlight the biological relevance and clarify what experiments would be needed to falsify our theories.

Our research started from questions in cognitive science (Schizophrenia and Charles Bonnet syndrome). The study became more theoretical after a lengthy search for models simple enough to yield formal understanding. Although abstract, we view this work as an important first step toward an information-theoretic understanding of pruning in biological networks.

Ultimately, Fisher information is a statistical property that can be computed from pairwise activity statistics. The same pairwise statistics exist in any spiking network, including biological ones. The measures we explore here therefore have a more general interpretation as a first-pass summary of the pairwise importance of correlated neural activity to generative representations. It is true that FI statistics may not correlate as closely with structural importance in biological networks, for many reasons. However, we expect the general ideas to be falsifiable.

We found that synaptic weights and local activity statistics provide a nuanced measure of synaptic importance, and that important synapses are associated with important neurons. This lays the groundwork to revisit and re-interpret experimental results. The most immediate prediction is that the probability of synapse elimination in a biological neuron should be a somewhat complicated function of its size as well as ongoing presynaptic and postsynaptic activity, and that this function should be connected to Fisher information in generative representations. We expect that the connection between local variables and statistics, and global network properties, is something that theorists and experimentalists will continue to explore.

To clarify biological relevance, we moved the discussion section on the biological plausibility of different pruning criteria forward (now lines 360-392) and augmented it with a paragraph where we discuss the suitability and biological detail of Boltzmann machines, starting from line 380. We also moved the whole discussion section forward to make it more central.

Reviewer #1, comment #2
Errors: It seems as if all the results stem from single network instances and there are no error estimates reported. As the pruning and RBM training heavily depend on the initial network, I think it would be good to compare results over multiple initialization.

Our response #1.2
Yes, we agree; We originally omitted multiple-trial validation because each simulation took several days to run. However, we were able to repeat ten trials for the results shown in figures 2-4. As you predicted, results vary depending on the random seed, but the overall results and conclusions are

the same. We updated Figures 2, 3, 4, and the associated discussion of these results starting line 152 for single layer RBMs and from line 219 for DBMs, to reflect these changes.

Reviewer #1, comment #3

Link to biology: The bidirectional weights and their training within RBMs are not exactly biological. Thus, it is unclear whether the advantage FI provides over weight-dependent pruning will still hold in biologically more realistic settings. Moreover, if the biological realistic implementation of an information based learning rule is in the focus, it should be highlighted and discussed better in the results instead of being hidden in the methods/appendix.

Our response #1.3

Indeed. We focused on RBM/DBMs in order to formally connect Fisher information to local statistics, and therefore provide rigorous mathematical results. The result that activity-driven plasticity causes weights themselves to relate to FI was an interesting surprise. This seems to imply that FI-pruning is quite accessible to biological networks (in some approximation). To make this more central, we now introduce and discuss the more biologically-plausible heuristic FI rule in the main text (line 137), rather than the appendix.

We are less concerned about the issue of symmetric weights or other details (like enforcing Dale's law). These details are of course important, but integrating them with the theory is beyond the scope of our study, which is ultimately the first steps of a larger theory. Fundamentally, the emerging dogma is that sensory systems learn spiking generative models of the world. Essential to this "predictive coding" is the idea that top-down feedback projections allow different layers of the biological neural network to exchange generative predictions consistent with bottom-up sensory input. We feel that the simplest theoretical model of this system is the DBM.

Naturally, however, biological networks must segregate feed-forward and feed-back connections into different weight matrices. Redundancy leaves considerable freedom for the feed-forward and feed-back weights to differ. However, feed-forward/feedback weights must generate self-consistent predictions. We therefore expect pairwise activity statistics to converge in biological systems to something similar to what we see in DBMs with symmetric weights (in some approximation). The FI-based measures of importance studied here are, ultimately, a function of these pairwise activity statistics.

It would be interesting to check, however, whether the heuristic-FI rule remains theoretically valid in the asymmetric case. These are very interesting directions of future study, but we do not have the capacity to extend the work further at this time.

As mentioned above (our response # 1.1), we adjusted the discussion section on the biological plausibility of different pruning criteria and added a more extensive discussion of the caveats incorporating these ideas (lines 368-392).

Reviewer #1, comment #4

Neuron removal: Throughout the paper, the authors often stress the fact that FI-based approaches prune connections such that whole neurons can be removed. I feel like there is a proper control missing for this to demonstrate the superiority of the FI approach. I would propose to introduce a pruning strategy which removes whole neurons (either randomly or by minimum sum of incoming weights). In that way, there would be a control case with similar neuron number to compare with.

Our response #1.4

Thank you for this suggestion for an additional control case. We included a pruning strategy that randomly removes hidden units while matching approximately the number of remaining weights.

We included this control case only for the pruning of our multi-layer DBM, since in the one-layer RBM all visible units are connected to all hidden units. When a hidden unit is removed here, the activity and Fisher information can completely re-arrange with re-training. This is not the case

for the multi-layer DBM configuration because here the second hidden layer depends on the first hidden layer. If the topology of the first hidden layer changes by random hidden unit removal, this may potentially damage the network in a way that it cannot recover from. This is what we observe: Random hidden unit removal irreversibly deteriorates performance, while the unit removal achieved by Fisher pruning reveals an efficient network architecture and maintains good performance until it is over-pruned.

Reviewer #1, comment #5
all Figures: Colors of Random and weight-dependent pruning were not so well discernible for me.

Our response #1.5
Yes, thank you; We changed the colors throughout and tried using an accessible color palette.

Reviewer #1, comment #6
p.2 l.81: it should be b^h in the parameter-set instead of h^h. Also, I would move the sentence about the Bernoulli RBM after the description of the activities, which are the binary quantities

Our response #1.6
Thank you! This is now fixed.

Reviewer #1, comment #7
p.3 l.87 I would move the discussion of diagonality after the example FI (Eqs.2/3), because i think these examples would make it easier to understand that non-local information is needed for the full FI

Our response #1.7
Thank you! We reworked this. Equations 2-3 now appear before the discussion of the full vs. diagnoal FIM, which starts at line 88.

Reviewer #1, comment #8
p.3 l.95 I am not completely sure what is meant by redundant here. Is each parameter redundant for the network or are they redundant (covariant or so) w.r.t. each other? Pleas clarify.

Our response #1.8
Agreed; The sentence was vague, and not central. We removed it.

Reviewer #1, comment #9
Fig 1C/D: the labels h and v were a bit confusing. Shouldn't it be b^v and b^h?

Our response #1.9
Indeed! This is now fixed.

Reviewer #1, comment #10
Fig 2: It is hard to see a strong effect in the pattern distributions. Is there a way to make this clearer? Possibly show ratios? Also the demonstrated changes in distributions will strongly depend on the size of the model and initial overparametrization

Our response #1.10
Thanks! This revealed a more serious issue: we were only looking at the rank-frequency plots for each model, which tell us something about the statistics of the model fit, but not much about how well the model is doing at predicting specific patterns. We replaced Figure 2 to show the effects on model accuracy more clearly. Even the "good" models allow a fair bit of variation in the

log-probabilities, but the differences in generative performance between the pruning rules remain clear.

Reviewer #1, comment #11
p.7 l 210 The explanation on generative performance is a bit distracting here. I would suggest moving it to the respective section.

Our response #1.11
Agreed; We moved this (now at lines 282-286) and reworked the text in the generative section.

Reviewer #1, comment #12
p.7 l 223 The reason for calculating the percentile is unclear. Are these the pruned synapses? Please clarify! Also, making more clear that these are the 10% of the weights with the lowest FI, would make sense.

Our response #1.12
We computed a percentile for each pruning criterion to find a threshold that restricts the pruning of synapses to $X\%$ of the current remaining synapses.

We changed this to read "On each iteration, we removed the least important 10% of weights as assessed by weight-specific FI or absolute weight magnitude in $\mathbf{h^1}$, and the least important 25% of weights in $\mathbf{h^2}$. For the FI-based rules, more than 25% of FI estimates of weights in $\vec{h}^2$ were zero in the first pruning iteration. In this scenario, we instead pruned all synapses with zero importance, which led to a comparably faster rate of model reduction."

We hope this is clearer.

Reviewer #1, comment #13
p.7 l.227 The reading flow here was a bit unsteady. Possibly move the statement of the before-pruning accuracy to line 210, where they are introduced.

Our response #1.13
Thank you, we moved this sentence earlier (now line 206-207).

Reviewer #1, comment #14
p.7 l. 236 Isn't this a consequence of your FI-estimator being zero for many more units than the weight such that you remove more connections. I think the plot 3b is a fair way to compare the pruning strategies. If you want to compare, let the weight-pruning run longer.

Our response #1.14
Thank you for this comment. We agree that the models should have a comparable number of weights when being evaluated. We now increased the percentage of removed weights in the second hidden layer from 10 to 25%. Since the FI-estimator was still zero for many weights (and as a consequence led to a faster reduction of units and weights), we present the FI results for fewer iterations than the other criteria in Figures 3 and 4. In appendix A3 we present the complete results for 10 pruning iterations for each criterion.

Reviewer #1, comment #15
Fig. 3C: Plotting over n_{w_h} does not add much here. I would plot over the iteration number for comparability.

Our response #1.15
Thanks for this suggestion. We think both versions have their advantages. We plotted the number

of units over the iteration number as you suggested (see Figure 1). But we eventually decided to keep the previous version of plotting over the number of weights. We want to highlight that while the number of weights are comparable, the number of units is not.

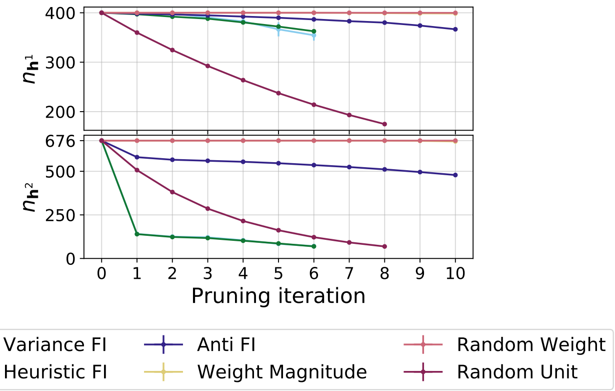

Figure 1: Number of units as a function of pruning iterations

Reviewer #1, comment #16
Fig. 4D: Why invert the x-axis?

Our response #1.16
Sorry about the confusion. We wanted to extrapolate the y-intercept when $n_{samples} \to \infty$. However, since the increase of the batch size from 1 to 10 for re-training generally improved digit diversity across criteria, we no longer investigated the effect of a full re-training.

Reviewer #1, comment #17
*p.9 l.311 *Anti-FI*

Our response #1.17
Thanks! We removed this since we no longer include the analysis of digit diversity after full re-training (digit diversity was not as adversely affected when allowing retraining with mini-batches of size 10).

Reviewer #1, comment #18
p.9 l.300 Typo "versus"

Our response #1.18
Thank you! This sentence was also removed due to drop of the analysis of digit diversity after full retraining from the results.

Our response #1.18
l. 353 There is a lot of experimental and theoretical work on connectivity overshoot, e.g. by Arjen van Oojen and Jaap van Pelt (based on activity dependent rewiring). Also spine turnover (especially removal) has been demonstrated to be much higher during maturation. Would this be the direction this is aiming at? What would be the functional use of a "critical phase"?

Our response #1.18
Thanks for suggesting this interesting work on activity-dependent structural plasticity! There is much more to be said here, and perhaps a current review is in order. However, we ultimately

removed this paragraph to make room for emphasizing biological relevance and caveats of our study.

(We had intended to conjecture that the optimal ”pruning rate” might vary over the course of learning, and that some schedules may be better than others (e.g. learning fully then pruning vs. pruning while learning). Some machine learning studies find that FIM plateaus at certain points during learning. Detecting this might provide an activity-driven and learning-related signal that can be used to modulate the rate of pruning. This is an interesting conjecture, but not central to the discussion of our main results.)

Reviewer #2, comment #1

A salient feature of neural development is pruning: the number of neurons and connections first grows, but at some point both decrease. It is not known why this happens, or which connections and neurons are pruned. The authors address the latter question, and propose that weights that have the smallest effects on activity, as measured by the Fisher information, are preferentially pruned; and if all connections from or to a neuron are pruned, the neuron is pruned as well.

I'm not an expert, but this is, I believe, a novel and interesting hypothesis, and will make a nice contribution to the field. My comments are almost exclusively about presentation – there were a lot of places I simply got lost. That didn't really detract from the big picture, but it would be nice if things were clarified.

Our response #2.1

Thank you, we are grateful for your feedback. We hope that the revised manuscript is more clear, and made changes throughout in line with your comments.

Reviewer #2, comment #2

Sloppiness is mentioned in the abstract, and not again until methods. It would make sense to me to either drop it in the abstract or mention it in the main text.

Our response #2.2

Thanks; We changed "sloppiness" to "importance"

Reviewer #2, comment #3

I personally would drop initials, and spell out RBM, DBM, FIM and FI. Or at least spell out the first three. I'm not sure why anybody uses initials; in my view authors should be allowed to use at most one. There's actually a reason for that: people rarely read papers beginning to end, and it's very annoying to have to hunt through a paper to look for the meaning of initials.

Our response #2.3

This is a good point. Expanding all abbreviations wasn't viable (the resulting text was too verbose). As a compromise, we now spell out Fisher information instead of FI, except when used to denote pruning rules like "heuristic FI" or "anti FI". We also frequently spell out deep Boltzmann machine when it appears without RBM in a sentence. We keep RBM and FIM throughout, but re-define them when they are used in figures, and periodically mention their full names at the beginning of sections. We replaced most instance s of "DBM" with the more general term "network". In the methods we mostly kept the abbreviations.

Reviewer #2, comment #4

Given the definition of the energy, I believe Eq. 2 is wrong: the derivative should be with respect to the partition function, not the energy. If so, that should be corrected.

Our response #2.4

We suspect that the definition in terms of the average curvature of the negative log-probability (i.e. energy) comes from statistics (as opposed to statistical physics). Assuming necessary regularity conditions, they are equivalent. First show that $\langle \nabla_\phi E \rangle = -\nabla_\phi \ln Z$:

$$\langle \nabla_\phi E \rangle = \int P \nabla_\phi E = \tfrac{1}{Z} \int e^{-E} \nabla_\phi E = \tfrac{1}{Z} \nabla_\phi \int e^{-E} = -\nabla_\phi \ln Z$$

Then from linearity it follows that $\langle \nabla^2_\phi E \rangle = -\nabla^2_\phi \ln Z$.

Reviewer #2, comment #5

I don't think P_v, h was ever defined.

Our response #2.5
Indeed, thanks! We now define this, stating "where $P_{\mathbf{v},\mathbf{h}}$ is the probability of jointly observing visible pattern **v** and hidden states **h**." (line 82).

Reviewer #2, comment #6
l 95-6: "When F_{ij} tends towards zero, the two parameters phi$_i$ and phi$_j$ are redundant." I can't for the life of me even guess what that means. It should be explained. Or dropped; I don't think it was ever used.

Our response #2.6
Indeed, we removed this. Thanks!

Reviewer #2, comment #7
l 99-100: "The resulting entries of the FIM depend on coincident firing of pre- and postsynaptic neurons and are arguably locally available." How can something that depends on two presynaptic and two postsynaptic neurons be locally available?

Our response #2.7
Thank you, this was poorly written. We meant to emphasize that the *diagonal* of the FIM is locally available. We have rewritten the explanation of local information in the diagonal of the FIM (lines 88-96) and hope this is clearer.

Reviewer #2, comment #8
I could not figure out what's in fig. 1c and d. This should be explained much more clearly.

Our response #2.8
Sorry for the poor explanation. We updated the caption to clarify, it now reads "(C): Importance of each parameter, as summarized by the value for each parameter in the vector of steepest curvature in the FIM (i.e. the leading eigenvector). The rectangular plot shows the normalized importance for all weights w_{ij} connecting visible units (v_i; vertical axis) and hidden units (h_j; horizontal axis). The importance for biases for the hidden ($\mathbf{b}^\mathbf{h}$) and visible ($\mathbf{b}^\mathbf{v}$) units is shown above (horizontal) and to the right (vertical), respectively. (D): Normalized parameter importance directly estimated from the diagonal entries of the FIM. (FIM = Fisher Information Matrix)"

Reviewer #2, comment #9
l 115-7: "The correspondence between parameter importance estimated from the first eigenvector and from the diagonal supports our use of Optimal Brain Damage for larger models, when computing the full FIM was no longer feasible." Couldn't make sense of this. It doesn't help that Optimal Brain Damage was (I believe) never explained.

Our response #2.9
Good point. There is no need to refer to "Optimal Brain Damage" in this location, except to note that Optimal Brain Damage also approximated the FIM with its diagonal. We removed the reference, and now only mention "Optimal Brain Damage" in the introduction when discussing prior work.

Reviewer #2, comment #10
l 117-22:. "Strikingly, the important weights typically aligned with few hidden units and their biases. This structure of the FIM suggests a separation into important hidden units and unimportant ones. It follows that FI motivated pruning likely leads to entire units becoming disconnected, which would allow their removal from the network. This would correspond to neuron apoptosis after excessive synaptic pruning." The second two sentences make sense. But the first two don't, and so it's not clear how the second follow from the first.

Our response #2.10
We changed this section to emphasize why the low-rank structure in the FIM implies that the (locally available) FIM diagonal is a good proxy for weight importance. We changed this section to better explain that important weights tend to be associated with connections to specific important units (lines 114-119).

Reviewer #2, comment #11
l 131-3: "For a pruning criterion based on the full FIM, we used the weight-specific entries of its first eigenvector as a direct indicator of weight importance." Couldn't make sense of this.

Our response #2.11
We changed this paragraph to read "We now present our results of applying the local estimate of Fisher information we introduced above as a criterion to prune RBMs. We compare this estimate to removing poorly specified, unimportant weights according to different pruning criteria and random pruning. We also test a pruning criterion based on the full FIM to confirm that the diagonal approximation closely tracks importance. For this, we assess importance as the magnitude assigned to each weight in the leading eigenvector of the FIM."

We also merged some of the discussion of the calculation of the FIM diagonal earlier in the text, where the FIM is first defined (lines 91-96).

Reviewer #2, comment #12
After Eq. 4, I think it's important to write down the expression for Fisher information (Eq. 19) that was actually used, written in a human-readable form. As far as I can tell, Eq. 19 can be written (... equation omitted ...) Algebra mistakes are possible, but I believe the correct expression looks something like this. And it's kind of easy to make sense of: besides the dependence on v_i and h_j, it's an approximately sigmoidal function of the weights. So it would be nice to include it.

Our response #2.12
This is very nice! We added the simplified equation to the main text (now Eq. 6), with some modifications to the text (lines 133-137). We hope you do not mind that we borrowed some of your wording in the appendix (see lines 760-770).

Reviewer #2, comment #13
l 160: "Generally, excessive pruning was detrimental to generative performance" It seems that it's only for the orange line (Heuristic FI?) that pruning was detrimental to generative performance.

Our response #2.13
We realized that the frequency plots were a poor choice for summarizing generative performance (our response # 1.10). Instead we now show the matched probabilities. In light of the updated results, the whole section was adjusted.

Reviewer #2, comment #14
Fig. 2 caption: "For all pruning strategies except Anti-FI pruning the model retains the ability to match the distribution of the training data (dashed lines) after retraining, indicating good generative performance." As far as I can tell, Anti-FI matched the true distribution in all but one panel.

Our response #2.14
Thank you, the revised Figure 2 fixes this. We now show the average error in the log-frequencies between the training data and sampled generated from the trained model. This shows that indeed the generated samples by an Anti-FI pruned model do not match the training data well.

Reviewer #2, comment #15
l 186-8: "In sum, for all pruning strategies (except Anti-FI pruning, which removed a large fraction of

important weights), the network could recover from the loss of weights and units through retraining". It looks to me like Anti-FI recovers as well.

Our response #2.15
Yes, although this was partly an artifact of the mistakes in plotting Figure 2. When plotting the matched probabilities of training instances and generated patterns it becomes evident that Anti-FI does recover, just not as well as other methods.

Reviewer #2, comment #16
mnist is 28x28. why scale to 20x20? The reason for this should probably be explained.

Our response #2.16
We removed the outer four pixels in order for the model to fit into the memory for our GPU. We now note this in the methods (line 451).

Reviewer #2, comment #17
Fig. 3B: why did random and [w] stop at 10^5*?*

Our response #2.17
The non-random pruning rules disconnect neurons that lose all of their incoming *or outgoing* synapses, thereby removing additional weights from the network. Since random pruning almost never disconnects whole neurons, we would have to run the simulation for significantly longer to simulate what happens when more weights are removed with random pruning.

In our new pruning schedule we therefore remove a higher percentage of weights from the second hidden layer to decrease the difference in remaining weights. To have a comparable number of weights, we show the FI pruning criteria for six iterations, while we let random pruning and the other criteria run longer.

Reviewer #2, comment #18
fig. 4: probability may not be the best measure – it's possible in principle that probability deteriorates but performance stays high. I think it would be good to report both performance and probability.

Our response #2.18
We show the classification performance in figure 3. We wanted to asses not whether a classifier can still function (MNIST is not a difficult task), but to assess whether the learned generative model was breaking down. To evaluate the generative performance, we came up with the two sub-tasks: the generated samples should resemble digits (quantified by a classifier's confidence) and they should be as diverse as the training data (quantified by the entropy over class counts when we let a classifier trained on the raw digits classify the samples). Since the re-training of models with mini-batches generally improved digit diversity, we redesigned the plot. We replaced figure 4B and removed figure 4D.

Reviewer #2, comment #19
And again, why stop at 10^5 *for random and [w], and even higher for Anti-FI?*

Our response #2.19
The reason was that the estimated Fisher information was zero for more than 10% of the weights, in which case we removed all of these 0-FI weights. However, we repeated the simulations. Although we still remove all 0-FI weights, we now let the weight/random/anti-Fi pruning run for more iterations to have a comparable number of remaining weights.

Reviewer #2, comment #20

l 350-3: "A recent study of the effects of perturbing the input during different time points of training in neural networks suggests that a critical learning period may be visible in a plateau of the FIM trace [45]." I couldn't make sense of this.

Our response #2.20

After consideration of both your and the other reviewer's comment on this paragraph, we decided to remove it (see our response # 1.18). (Our results do not directly address critical periods and we wanted to make room for the discussion of biological relevance.)
