# Supplementary material for: The information theory of developmental pruning: Optimizing global network architectures using local synaptic rules
Source: PLoS Comput Biol. 2021 Oct 11;17(10):e1009458. doi: 10.1371/journal.pcbi.1009458 (PMC8584672; doi:10.1371/journal.pcbi.1009458)
Supplement: Attachment — Submitted filename: rebuttal_letter2_ploscomp.pdf [file pcbi.1009458.s004.pdf]

Reply to the Reviewers

Re: Manuscript ID PCOMPBIOL-D-20-02149
"The Information Theory of Developmental Pruning: Optimizing Global Network Architecture Using Local Synaptic Rules"
Carolin Scholl, Michael E. Rule, and Matthias H. Hennig
PLOS Computational Biology

Dear Reviewers and Editors,

Thank you for your consideration and for the opportunity to submit a second revised draft for publication in PLOS Computational Biology. We are very grateful for the feedback and ideas for improvement.

We have taken the reviewer feedback into account and revised the manuscript accordingly. All changes are highlighted: blue indicates added content and red indicates deleted content. Please see the point-by-point response to the reviewers' comments and concerns below. All page and line numbers refer to the newly revised manuscript file.

We hope that the revised manuscript will remedy all concerns, but are happy to consider further revisions, and we thank you again for your continued interest in our research.

Yours sincerely,
Matthias Hennig, Michael Rule, and Carolin Scholl

Reviewer #1, comment #1

The authors put great effort into improving the paper and addressed most of my concerns. I only have a few remaining issues (and suggestions) before I recommend publication:

Concerning 1.4: Random unit pruning: (a) "... in the one-layer RBM all visible units are connected to all hidden units. When a hidden unit is removed here, the activity and Fisher information can completely re-arrange with re-training". As the unit pruning seems to be the main advantage of the FI-approach, I think this control case should also be included for the single layer RBM. The above intuition can the be discussed and demonstrated in Fig 2C: One would see a large divergence before but not after retraining.

Our response #1.1

We had conducted this experiment, and initially decided not to include it in the original manuscript. The results are as you predicted: a large divergence before, but not after retraining. We added this result in Figure 2C of the revised manuscript. We further added a paragraph where we discuss this additional control case (see Lines 181-189).

Reviewer #1, comment #2

(b) From what I see in Figure 3, the random unit pruning is not really fair with respect to the units in hidden layer 1, as it removes an order of mangitude more neurons in that layer. To really demonstrate that FI-pruning leads to a better network structure more quickly, I propose to adapt this and remove less neurons in h1 to arrive at a comparable structure

Our response #1.2

Indeed the number of units in hidden layer 1 is much lower in the case of random unit removal. The random unit removal was included as an additional control case after the first revision. We implemented it in such a way that a comparable number of weights is pruned as with our synaptic pruning rules. We think it is a suitable control to show that FI-pruning allows topological optimization of the network in the different layers as it preserves more units in the first layer (see lines 277 - 282).

Since the ratio of weights removed to units removed is fixed for "random unit" pruning, it is impossible to match the horizontal axes between all plots in F3B,C. We focus on how pruning of weights can optimize the network, with unit-pruning as a useful emergent side-effect of using the FI-based rules. If one were to remove fewer units in Figure 3C (top), such that the random unit pruning removes the same number of units, then the number of weights would not be matched in Figure 3B.

Still, we conducted another experiment where we removed less units in the first hidden layer (corresponding to 5% of weights instead of 10%). Even then, more hidden units were removed than with the other criteria. As one can see in panel B of Figure 1, the layer ends up with a higher number of weights than when it was pruned according to other criteria, complicating comparability.

The encoding performance deteriorated to a similar degree (see Figure 1). It demonstrates that the first hidden layer is a bottleneck for performance: if it loses too many neurons, the performance decreases.

Reviewer #1, comment #3

l.134/l.333f Could you provide more motivation why it is easier to track firing rates and weights instead of correlation and weights. Biologically, Ca or CaMKII are thought to be local proxies of correlated activity, but I am not aware of molecular signals tracking especially the presynaptic rate.

Our response #1.3

This is true. We think it is interesting to note that weight magnitude (i.e. synaptic strength) can serve as a proxy for these correlations, since it allows for a various possible biological implementations. Experiments to explore whether similar pruning rules occur *in vivo* should therefore explore

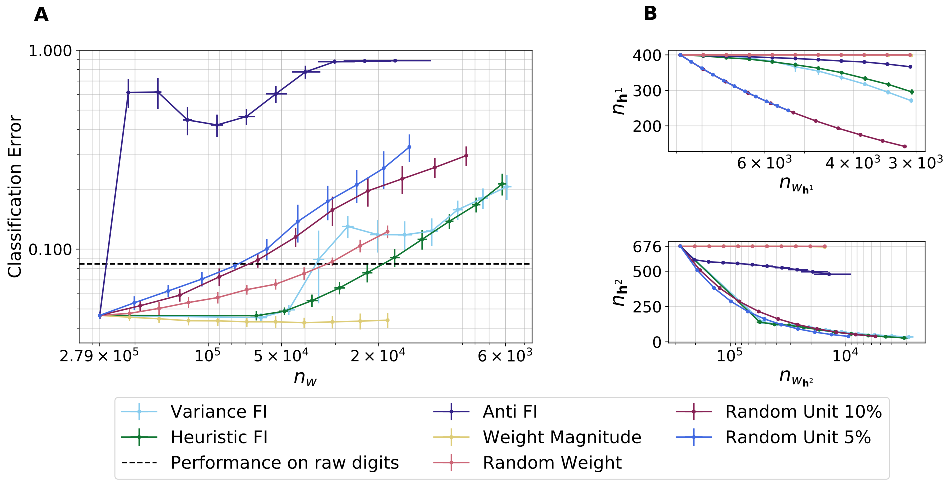

Figure 1: Figure 3 including a simulation where 5% of weights were removed by random unit removal in the first hidden layer (as opposed to 10%).

not just connections to correlations, but other variables that correlate with synaptic weights. We now briefly elaborate on this in Lines 135-139.

Reviewer #1, comment #4

l.165 I guess here you need to discuss the results a bit deeper, as otherwise panel 2C would have been sufficient to make the point. Specifically, I noticed that the generative performance only seems to be poor for seldom patterns whereas the performance for abundant patterns seem to match (although with larger variation in the Anti-FI case). Is this really so bad for neural system? From an information theoretic viewpoint, they are surely the most informative patterns. However, as these unmatched patterns are rare, the error introduced by them may be negligible.

Our response #1.4

Thanks for this suggestion. This is an issue with most theoretical neuroscience works that explore sensory channels as optimal encoders. Not all sensory information is equally important, and in practice sensory systems do not transmit all patterns. The selection of information however likely also depends on ecological and behavioural factors, and it seems difficult to test hypotheses beyond a general information maximisation objective. The energies in our models here could be interpreted not as the environmental probabilities, but rather a more complicated behavioral cost function. To model this explicitly, one would need to modify the wake-sleep learning rules to adjust pattern frequency or the amount of plasticity driven by each pattern. This is an interesting line of further study, but beyond the scope of our work.

Yet we agree that we should discuss Figure 2B further. We briefly explain the results that can be seen in the figure now (Lines 175-178).

Reviewer #1, comment #5

l.372 The statement seems a bit bold. Maybe use "activity-dependent pruning that aims to identify uninformative neurons"

Our response #1.5

We agree and changed the sentence to read as suggested.

Reviewer #1, comment #6

Suggestions to improve readability:

- In my opinion, it would make sense to move the introduction of the RBMs (l.23-33) to the end of the introduction (after l.55)

Our response #1.6
Thanks for the suggestion. We agree that moving this part to the suggested position eases the flow of reading. We adjusted the text accordingly.

Reviewer #1, comment #7
- l.70 Maybe one could also mention the relation between energy and pattern probability in equation 1.

Our response #1.7
Thanks, we added the sentence "Lower energy corresponds to higher probability of the respective model state." at line 74.

Reviewer #1, comment #8
- l.101 I would mention how the models were fitted here (wake sleep algorithm).

Our response #1.8
Thanks! We added this (now at line 102).

Reviewer #1, comment #9
- l.101 It is not immediately clear what is meant by "parameter-wise" (first mention). I would stick to the terms full and diagonal or at least specify what is meant in this sentence. Moreover, I think it is may be less confusing to discuss the results in the order they are presented in the figure and move the Also, an activity dependent form is only available from Equation 3 or 4, right?

Our response #1.9
Thanks a lot for this comment. We agree that the flow of reading was a bit unsteady here. We changed the order to match the one one presented in the figure. We also dropped the term parameter-wise throughout the manuscript and no longer refer to Equation 2 here. Thanks for noticing this!

Reviewer #1, comment #10
- l.141 It is not immediately clear why the FI introduced before is "variance" based. Maybe the term could be introduced together with the method and the motivation of "variance" could be explained.

Our response #1.10
Thanks, we now explain why we call it the variance estimate of FI in the paragraph above the introduction of the heuristic estimate (see lines 133-134).

Reviewer #1, comment #11
- l.150 I think it should be shortly motivated what the generative performance means/relates to in the neuronal/biological system, to give a better intuition what the FI-approach actually preserves.

Our response #1.11
In RBMs and DBMs, good generative performance is equivalent to Shannon-optimal encoding (Hinton et al. 1995). It also implies internal models that can accurately predict lower-level inputs from internal states. We now motivate evaluating the generative performance and comment on this in the text at lines 152-157.

Reviewer #1, comment #12

Finally, I would have another suggestion: Another advantage of the FI-dependent pruning over other methods may be the fact that it could be used to determine when pruning should be stopped. At the moment this is not the case as the lowest-FI quantile of synapses is always removed. If, instead, only synapses below an FI-threshold would be removed, pruning would naturally stop if all synapses have high FI. Such a convergence would remove the necessity to select a suitable number of pruning iterations for the model and prevent the performance loss of the FI-based models after massive pruning in Fig 3. Assuming that pruning stops after all synapses have high FI, one would get one "optimal" pruned model (instead of one per pruning iteration). Determining these optimal models for different input statistic would also allow predictions on the number of surviving synapses and neurons as well as weight distributions (for example comparing the networks after training with a 5-class MNIST subset and the full dataset). Varying the input statistics and getting different resulting models would greatly underline the point that FI-pruning actually selects input-related "optimal" model architectures and not just "smaller" models whose size is determined by the number of iterations. Moreover, such an analysis would provide more insight into the relation between the encoding of the Boltzmann machine and optimal pruned models, which, I guess, was a goal of this line of research. The differences in the resulting optimal networks could, in turn, be compared with existing data on network complexity/ neuron and spine densities in animals reared in different environments (e.g. dark rearing, rearing with differently oriented bars, normal cages, enriched environments). This would make a nice connection to biology and provide actually testable pre/postdictions (Concerning the experiments you proposed: at least the experimentalists I know say that it is not feasible to track pre- and postsynaptic activity and the weight of an identified synapse over time at the moment).

I am aware that this additional analysis may be work-intensive and beyond the scope of this paper. However, I think it may greatly improve the manuscript or at least provide an interesting direction for future research.

Our response #1.12

These are really interesting thoughts and ideas for future directions. We included an additional graph in the appendix of the previous revised manuscript, showing that a rise in the average latent activity may also be a signal to stop pruning.

Unfortunately we have to agree that these analyses are out of the scope of this article. However, they will be an excellent project for future students.

Reviewer #2, comment #1
The paper is much improved, and I'm happy with it. Only two comments:

1. in Eq. 10, I believe the weights should have superscripts.

Our response #2.1
You are right, thank you for noticing this. We introduced the weight matrix $\mathbf{W}^{\mathbf{h}^2}$ afterwards, but did not use the superscripts in Eq. 10. This is now fixed.

Reviewer #2, comment #2
2. I would suggest moving A1 and A2 to Methods. I suspected the more mathematically inclined will be interested. I certainly was, since I got it wrong the first time around. ;) This is, though, completely up to the authors.

Our response #2.2
Thanks for the suggestion. We agree that A1 and A2 rather belong in the methods instead of the supplementary material. We moved the parts accordingly.
